# Efficacy of Inhalational Ciprofloxacin (Apulmiq) in an NHP Model of Q Fever

**DOI:** 10.3390/antibiotics15080773

**Published:** 2026-08-11

**Authors:** Michelle Nelson, Rachel E. Ireland, Francisco J. Salguero, Stuart J. Armstrong, Thomas R. Laws, James D. Blanchard, Francis Dayton, Igor Gonda, Chad W. Stratilo

**Affiliations:** 1 Defence Science and Technology Laboratory, Porton Down, Salisbury SP4 0JQ, UK; reireland@dstl.gov.uk (R.E.I.); sjarmstrong@dstl.gov.uk (S.J.A.); trlaws@dstl.gov.uk (T.R.L.); 2 UK Health Security Agency, Porton Down, Salisbury E14 4PU, UK; javier.salguero@ukhsa.gov.uk; 3 Aradigm Corporation, Hayward, CA 94545, USA; sweeneytds@gmail.com (J.D.B.); francisdayton@outlook.com (F.D.); igonda@respidex.com (I.G.); 4 Bio Threat Defence Section, Suffield Research Centre, Defence Research and Development Canada, Medicine Hat, AB T1A 8K6, Canada; chad.stratilo@drdc-rddc.gc.ca

**Keywords:** *Coxiella burnetii*, Inhaled antibiotic therapy, respiratory infection, marmoset, *Callithrix jacchus*, antimicrobial therapy, doxycycline

## Abstract

Background/Objectives: Q fever, caused by *Coxiella burnetii*, is a zoonotic disease usually treated with doxycycline, but alternative options are needed when doxycycline is unsuitable. This study evaluated inhaled liposomal ciprofloxacin (Apulmiq^®^) as a post-exposure prophylaxis (PEP) compared with oral doxycycline in a common marmoset model. Methods: Marmosets were exposed to aerosolised *C. burnetii* and treated 24 h later with Apulmiq (0.8 mg/kg inhaled), doxycycline (6 mg/kg oral), or placebo for 7 days. Outcomes included fever, weight loss, bacterial burden, histopathology, liver enzymes, and immune responses. Results: Both treatments delayed fever onset and reduced disease severity versus placebo. Doxycycline prevented fever during treatment and significantly reduced bacterial loads in the lungs and spleen by Day 28. Apulmiq slightly delayed the onset of fever and reduced early bacteraemia but was less effective at bacterial clearance. Immune analyses showed macrophage activation and elevation of IFN-γ during acute infection, and adaptive immune responses by Day 28. Conclusions: Inhaled Apulmiq provided partial prophylactic protection but did not achieve bacterial clearance. Doxycycline reduced bacterial burden but did not completely eliminate disease. Inhaled antibiotics may have potential for use as an alternative approach for PEP treatment, although further optimisation and investigation are needed.

## 1. Introduction

Q fever is a zoonotic disease caused by the Gram-negative, intracellular bacterium, *Coxiella burnetii.* The disease is almost ubiquitous throughout the world and is predominantly associated with farm animals, such as cattle, sheep, and goats. Spread to humans usually occurs by the aerosol route, typically in people living in close proximity to infected livestock [1,2]. Human Q fever can be asymptomatic or self-limiting. The 40% of individuals who develop the acute disease, predominately present with a fever, headache, malaise, and atypical pneumonia, although cough and hyperhidrosis can be common [3]. Typically, 1–5% of cases develop into a chronic form of the disease, which is usually associated with heart valve issues, in immunocompromised people or pregnant women.

Doxycycline remains the first line of antimicrobial defence for Q fever, with twice-daily administration of 100 mg of antibiotic recommended [4]. Doxycycline inhibits bacterial protein synthesis by binding to the 30 s ribosomal unit, which prevents bacterial multiplication without killing the bacteria (bacteriostatic). In addition to its use for Q fever, it is also used to treat a range of bacterial infections including urinary tract infections and sexually transmitted diseases and to treat acne and certain tick-borne diseases. Alternative therapies are required for children and pregnant women and when there is intolerance to doxycycline [5]. There is a link between the development of chronic Q fever and mismanagement of treatment [6]. Delivery of antibiotic directly to the lungs, the primary route of entry of *C. burnetii*, has the potential to prevent systemic spread of the bacteria and reduce the development of chronic disease. Liposomal encapsulated formulations of ciprofloxacin have been developed for inhalation delivery. Lipoquin^®^ and Apulmiq^®^ were developed by Aradigm Corporation [7,8], and have undergone Phase III human clinical trials for non-cystic fibrosis bronchiectasis and chronic lung infections with *P. aeruginosa* [8]. Apulmiq is a dual-phase delivery of liposomal encapsulated and free ciprofloxacin, providing an immediate high concentration of antibiotic in the lungs followed by sustained, slow release that goes systemic. Ciprofloxacin enters bacterial cells and inhibits DNA gyrase and topoisomerase IV enzyme. Both Lipoquin and Apulmiq have been evaluated against inhalational tularaemia, plague, anthrax, and Q fever infections in mice [9,10,11,12,13]. Subsequently, the utility of Apulmiq^®^ in both prophylaxis and treatment of tularaemia in a marmoset model has been determined [14].

The common marmoset (*Callithrix jacchus*) has been used to characterise a number of infectious diseases, including Q fever [15]. The marmoset model of Q fever is highly representative of the disease in humans. Marmosets have a resolving febrile response, typically between 4 and 11 days, depending on the challenge dose. A Q fever study in marmosets reported that infection was also associated with weight loss between 5- and 15-days post-challenge, liver enzyme dysfunction, and elevated expression of CD64 (sepsis marker) on circulating neutrophils during bacteraemia. Bacteraemia and circulating IFN-γ were evident from Day 7 post-challenge, resolving by Day 21. *C. burnetii* was recovered from the lungs of 75% of the animals at Day 28, when there were no other overt clinical features of disease. Histologically, there was evidence of macrophage and lymphocyte infiltration into the lung resulting in granulomatous alveolitis. Febrile disease, fatigue, headache, myalgia, and pneumonia with or without hepatitis are key features of the human disease [3].

The aim of this study was to assess whether inhalation delivery of Apulmiq is a superior post-exposure prophylactic (PEP) option to orally delivered doxycycline, using a nonhuman primate model of human disease.

## 2. Results

### 2.1. Pharmacokinetic Parameters of Doxycycline in Naïve Marmosets

In order to determine a human-equivalent dose of doxycycline in the marmoset, two studies were performed: a single-dose study and a multiple dose study. The pharmacokinetic parameters obtained following a single oral dose of 10 mg/kg of doxycycline were used to perform non-compartmental analysis (Table 1). The Area under the Curve (AUC) was 64.9 h.mg/L, which was higher than the target AUC of between 13 and 40 h.mg/L [16]. Prediction modelling was performed to determine a more appropriate dosing regimen for the multi-dose study that achieved the target AUC. This was based on a linear relationship in response.

Various dosing regimes were modelled from the 10 mg/kg empirical data looking at different concentrations of antibiotic and the frequency of dosing. For a dosing regimen of 4 mg/kg, administered twice a day at 12 hourly intervals, the AUC was predicted to be 33.2 h.mg/L. Once-daily dosing of 4 mg/kg of doxycycline was predicted to give an AUC of 20.1 h.mg/L. Both dosing regimens are within the desirable human range; however, the once-daily dosing AUC was equivalent to the dose used in murine studies [11]. In both cases, the levels of antibiotic were maintained above the minimum inhibitor concentration (MIC) of doxycycline against *C. burnetii* for the duration of dosing (0.01 to 0.04 μg/mL) [17]. Therefore, a dosing regimen of 4 mg/kg once a day was selected for the multiple dose study.

**Figure 1:**
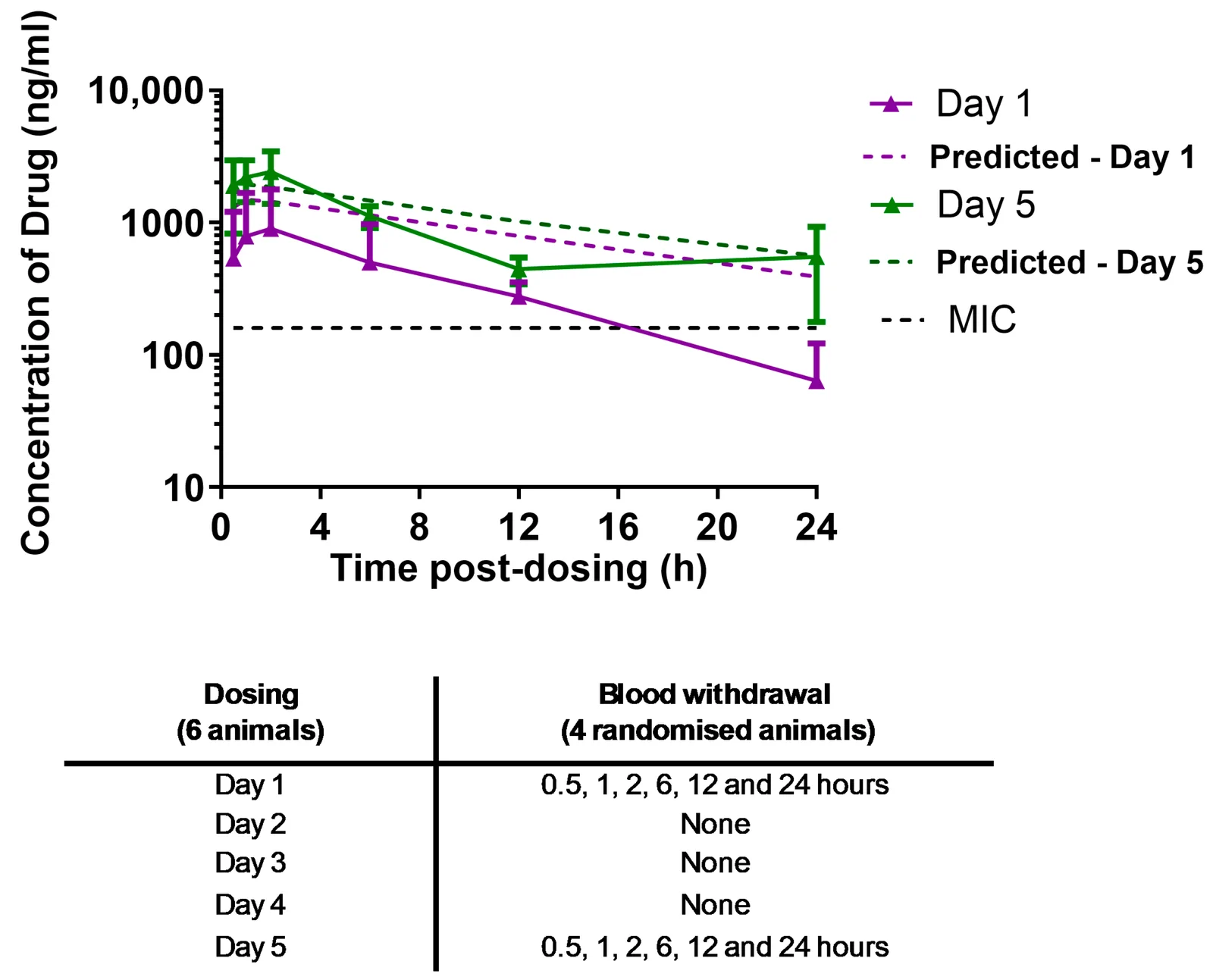
Concentration of doxycycline in the plasma of marmosets following dose 1 (Day 1) or Dose 5 (Day 5) of 4 mg/kg administered at 24 h dosing intervals. Dashed green and purple lines indicate the predicted profile using non-compartmental modelling of the data following a single dose of 10 mg/kg. Error bars represent the standard error of the mean of 4 randomised animals from the pool per timepoint.

In the multiple dose study, the concentration of doxycycline in the plasma was determined following the administration of multiple doses of 4 mg/kg of antibiotic (Figure 1). The T_max_ was similar after the first and fifth dose, of 1.8 and 1.4 h, respectively (Table 1). However, the C_max_ (0.9 and 2.4 mg/L) and AUC (8.4 and 17.5 h.mg/L) were strikingly different between the first and fifth doses. This was despite the t_1/2_ being similar at 5.2 and 4.0 h. One-compartment modelling was able to fit the data at Day 1 and Day 5 separately. However, one-compartment modelling of data from Day 1 would not fit the data from Day 5. No other model was able to fit the data. Additionally, correction of the AUC and C_max_ based on the dose to standardise the data should give similar values for data with a linear relationship. However, this is not true for the data obtained in this study. A non-linear relationship would explain the inability to model the data accurately. Further to this, the expected accumulation index is 1.05, where the elimination rate constant (k) and the dosing interval (τ) are 0.13 1/h and τ = 24 h, respectively. However, the observed accumulation index is 2.25, further indicating non-linearity.

Based on the data above, the dose selected for the efficacy study was pragmatically selected as 6 mg/kg, without the need to use any further animals. The 4 mg/kg dosing regimen resulted in a lower than anticipated AUC after both 1 and 5 doses. However, the AUC following administration of 10 mg/kg was higher than the target human dose of 13 to 40 h.mg/L [16]. Therefore, a dose slightly higher than 4 mg/kg was required to increase the AUC after 1 dose whilst accounting for the higher AUC that occurred after 5 doses. A conservative increase to 6 mg/kg was rationally selected, which predicted an AUC at the lower end of the target range, also providing a safety margin when extrapolating to human data. The pharmacokinetic parameters were not measured in infected animals as part of the efficacy study.

### 2.2. A Comparison of the Efficacy of Apulmiq and Doxycycline Prophylaxis Against Q Fever

Marmosets were challenged with *C. burnetii* by the aerosol route. The actual challenge dose each animal received was based on the volume of air that the animal breathed during the challenge, with a mean challenge dose of 3.2 × 10^6^ ± 1.1 × 10^6^ cfu, ranging from 9.0 × 10^5^ cfu to 4.2 × 10^6^ cfu. There was no significant difference related to the challenge dose received by each cohort of animals. At 24 h post-challenge, doxycycline (6 mg/kg) or Apulmiq (0.8 mg/kg) [14] administration was initiated by the oral or inhalation routes, respectively, and continued once a day for 7 days. A 30 min exposure of marmosets to Apulmiq that resulted in a human-equivalent target dose of 0.8 mg/kg body weight in the lungs was previosuly reported [14]. Negative control groups received vehicle placebo or empty liposomes by the oral or inhalation routes, respectively, at the same time. The disease was monitored in terms of the temperature response, weight loss, lung pathology at Day 28, liver enzyme levels, and the bacteriology and immunological responses.

**Figure 2:**
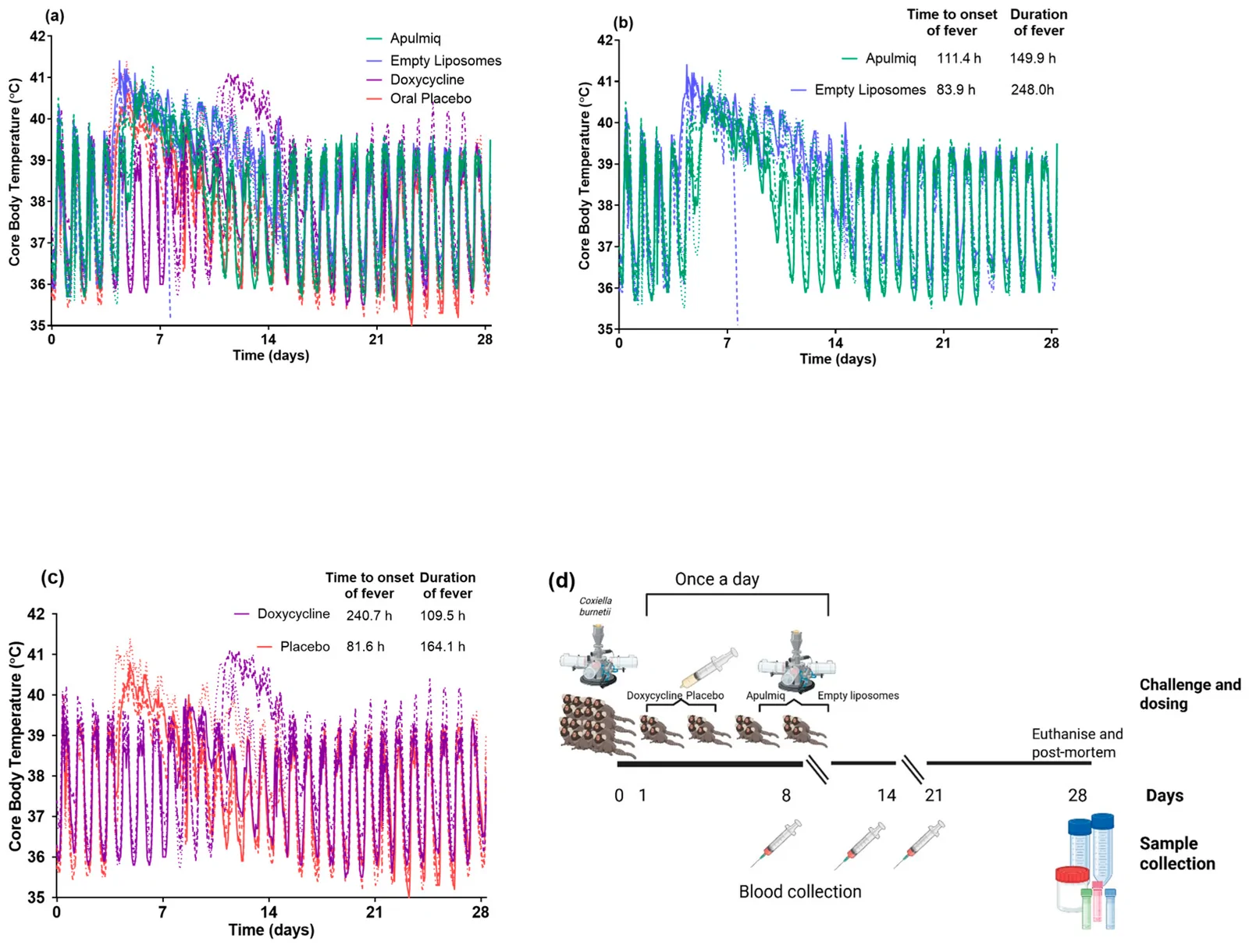
Temperature profile of marmosets challenged with *C. burnetii* and administered therapy by different routes. (**a**) Summary of all animals. (**b**) Comparison of animals administered therapy or placebo by the inhalation route. (**c**) Comparison of animals administered therapy or placebo by the oral route. (**d**) Schematic of challenge and dosing regimen (Created in BioRender. Scott, A. (2026) https://BioRender.com/bsfnmbb, accessed on 3 August 2026). Each group of animals is represented by a different colour line (green—Apulmiq, blue—empty liposomes, purple—doxycycline, and red—oral placebo), with the line of each of the 4 animals per group having a different pattern of dashing.

All animals maintained their normal diurnal rhythm for at least 3 days (80 h) post-challenge when the placebo animals became febrile (defined as greater than 40 °C for at least three consecutive readings) (Figure 2). Animals that received oral placebo became febrile at a mean time of 81.6 ± 1.5 h post-challenge. Animals that received inhalational placebo (empty liposomes) became febrile at a mean time of 83.9 ± 3.0 h post-challenge. Animals remained febrile for a total of 164.1 ± 27.5 and 248.0 ± 37.8 h post-challenge, respectively, prior to the fever resolving. However, the fever resolved in two phases. Initially, the animals’ temperature returned to a normal daytime temperature (i.e., was less than 40 °C), but still remained at least 1 °C higher than normal during the nighttime phase, for up to four additional nights. The temperature of one animal that received empty liposomes began to decline rapidly from 7 days post-challenge; this animal became reluctant to move and hunched, and was euthanised on Day 8 due to their rapidly declining condition. All other animals survived until the end of the study with no overt clinical signs.

Animals that received Apulmiq had a statistically significant delay in the onset of fever compared to those that received empty liposomes (*p* < 0.05), with the onset of fever occurring at 111.4 ± 10.1 h post-challenge. The animals that received doxycycline remained afebrile throughout the therapy window. However, following cessation of antibiotic, all animals that received doxycycline had a raised temperature. One animal had a raised temperature during the nighttime phase for approximately 3 nights, whereas the remaining three animals all developed a temperature above 40 °C for between 34 to 78 h (mean of 40.3 ± 16.2 h). The onset of fever was statistically significant later than the oral placebo group (*p* < 0.001) and the Apulmiq group (*p* < 0.001).

**Figure 3:**
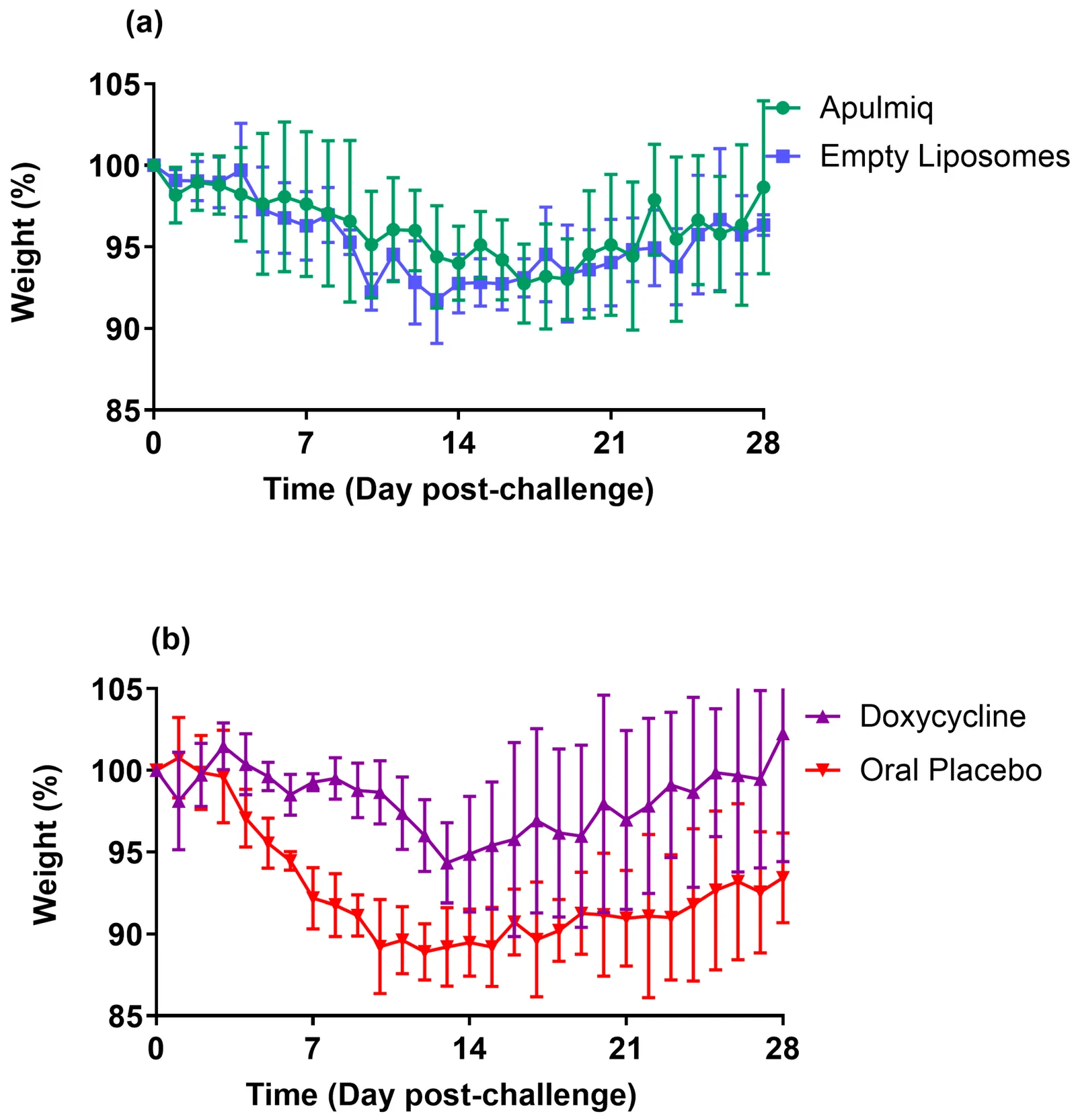
Change in weight observed in animals challenged with *C. burnetii* and administered therapy once a day for 7 days. (**a**) Mean weight change for animals given therapy by the inhalation route. (**b**) Mean weight change for animals given therapy by the oral route. There are 4 animals per group and the error bars represent the standard deviation.

Animals were weighed on a daily basis during the study. In general, all animals lost up to a maximum of 12% of their body weight, starting from Day 3 post-challenge (Figure 3). Animals that received antibiotic, either Apulmiq or doxycycline, lost less weight compared to the control animals, empty liposomes, or oral placebo. The peak of the weight loss for the oral placebo animals was on Day 12, where the mean loss was 11.1 ± 0.9%. The peak weight loss for the empty liposomes and doxycycline groups was on Day 13, where they lost 8.2 ± 1.3% and 5.7 ± 0.9%, respectively. There was a delay in the peak weight loss in the Apulmiq group, which lost 7.3 ± 1.2% on Day 17. The weight of all animals was returning to baseline values by the end of the study (Day 28), although the doxycycline group exceeded this value.

**Figure 4:**
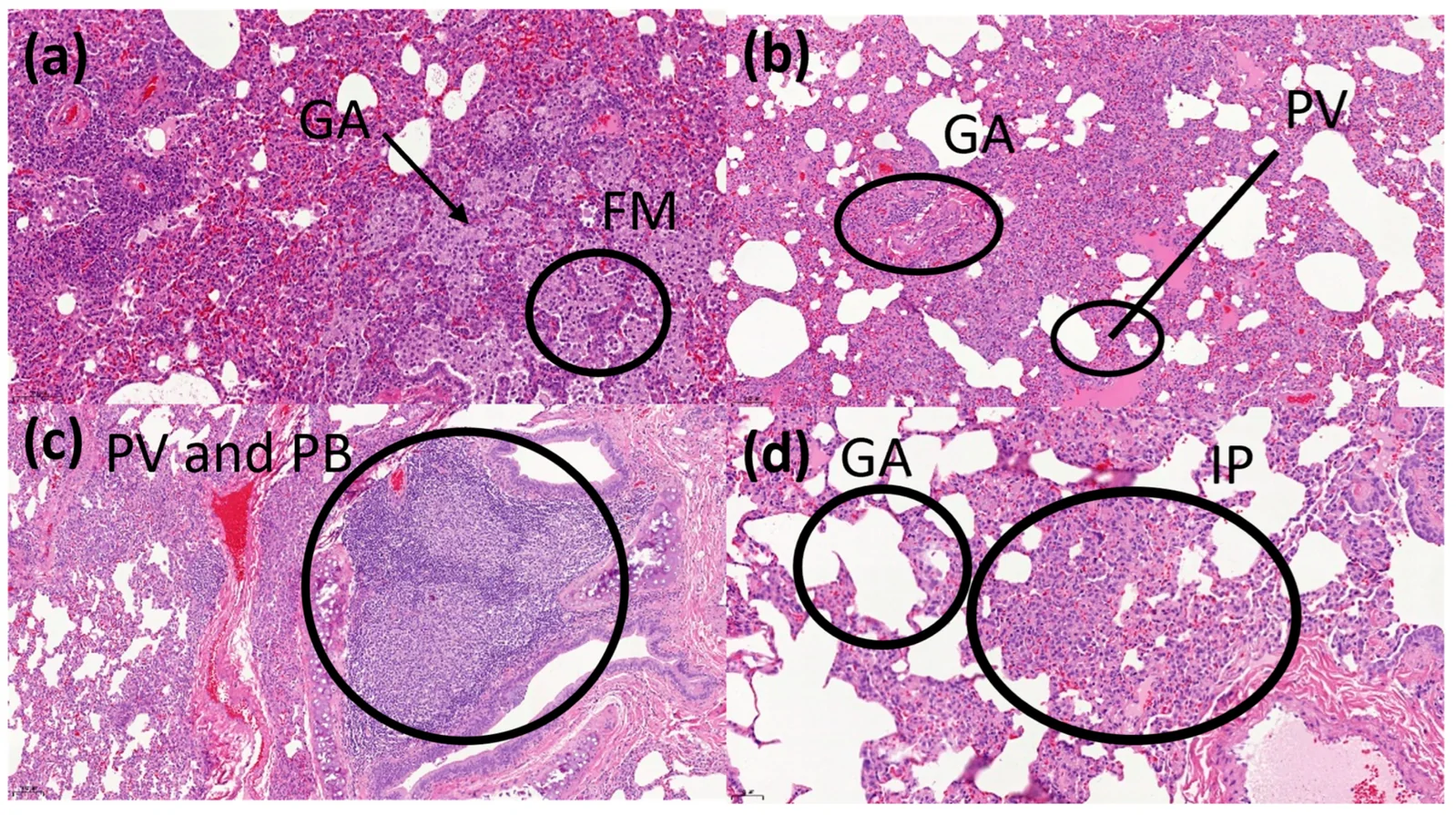
Representative H&E-stained lung sections from marmosets following challenge with *C. burnetii* and administered therapy by either the inhalation or oral routes. (**a**) Section from an animal that received empty liposomes with severe granulatomous alveolitis (GA) and foamy macrophages (FMs). (**b**) Section from an animal that received Apulmiq with moderate granulomatous alveolitis (GA) and perivascular cuffing (PV). (**c**) Section from an animal that received oral placebo with severe perivascular (PV) and peribronchiolar (PB) cuffing. (**d**) Section from an animal that received doxycycline with mild granulatomous alveolitis (GA) and interstitial pneumonia (IP).

Tissue samples collected during the post-mortem of animals were analysed to determine any cellular morphology changes. This included the one animal that received empty liposomes and was euthanised because they reached the humane endpoint and the remaining 15 animals that survived to the end of the study. Disease-related pathological features were observed in the lungs of all animals (Figure 4). In general, the total severity of lesions in the lung was greater in the control animals (empty liposomes or oral placebo), with total severity scores of 15.8 ± 3.5 and 13.0 ± 5.0, respectively. The least severe lesions were observed in animals that received doxycycline; however, the severity was only statistically significantly lower than the lesions observed following delivery of empty liposomes (*p* = 0.01). The most common severe finding was the presence of moderate to severe granulomatous alveolitis, which had a mean severity score of 3.5 and 3 for oral placebo and empty liposomes, respectively. Granulomatous alveolitis was mild to moderate in the animals that received antibiotics, with mean severity scores of 2.75 and 2 for animals given Apulmiq and doxycycline, respectively. Moderate to severe perivascular and peribronchiolar cuffing was also observed in the control animals (severity scores of 3 and 2.75 for both of the control groups). In addition, mild to moderate pyogranulomatous alveolitis and moderate acute foci bronchiolitis was also observed in animals that received empty liposomes. A minimal to mild presentation was observed in animals that received antibiotics. Interstitial pneumonia and vasculitis were minimal to mild in all groups.

Generally, the pathological features observed in the other organs, liver, spleen, and kidney were background findings apart from the severe inflammatory infiltrates in the liver of one animal that received empty liposomes and the kidney of one animal that received doxycycline. The minimal to mild lymphoid depletion in the lymph nodes may be indicative of an active immune response. Interestingly, one animal that received empty liposomes had inflammatory cell infiltration in the myocardium/epicardium.

The level of bacteraemia was assessed on Days 8, 14, and 21 post-challenge. On Day 8 post-challenge, higher levels of bacteraemia were observed in the control animals (empty liposomes and oral placebo), with mean levels of 650 ± 70 cfu and 245 ± 88 cfu, respectively. Three out of four animals that received Apulmiq had bacteraemia (mean values of 30 ± 17 cfu), compared to one out of four animals that received doxycycline (20 cfu). All animals that were febrile also had bacteraemia, except one animal that received Apulmiq, where no bacteria were detected in the blood. The animal that received doxycycline and had a low level of bacteria in the blood, had a raised body temperature at night. By Day 14 post-challenge, the level of bacteria in the blood had decreased in all groups of animals except those that received doxycycline, which increased to 70 ± 47 cfu (2/4 animals had bacteria in their blood). This increase in bacteraemia was associated with the delayed fever observed in this group of animals. No bacteria were recovered from the blood of animals at Day 21 or Day 28.

**Figure 5:**
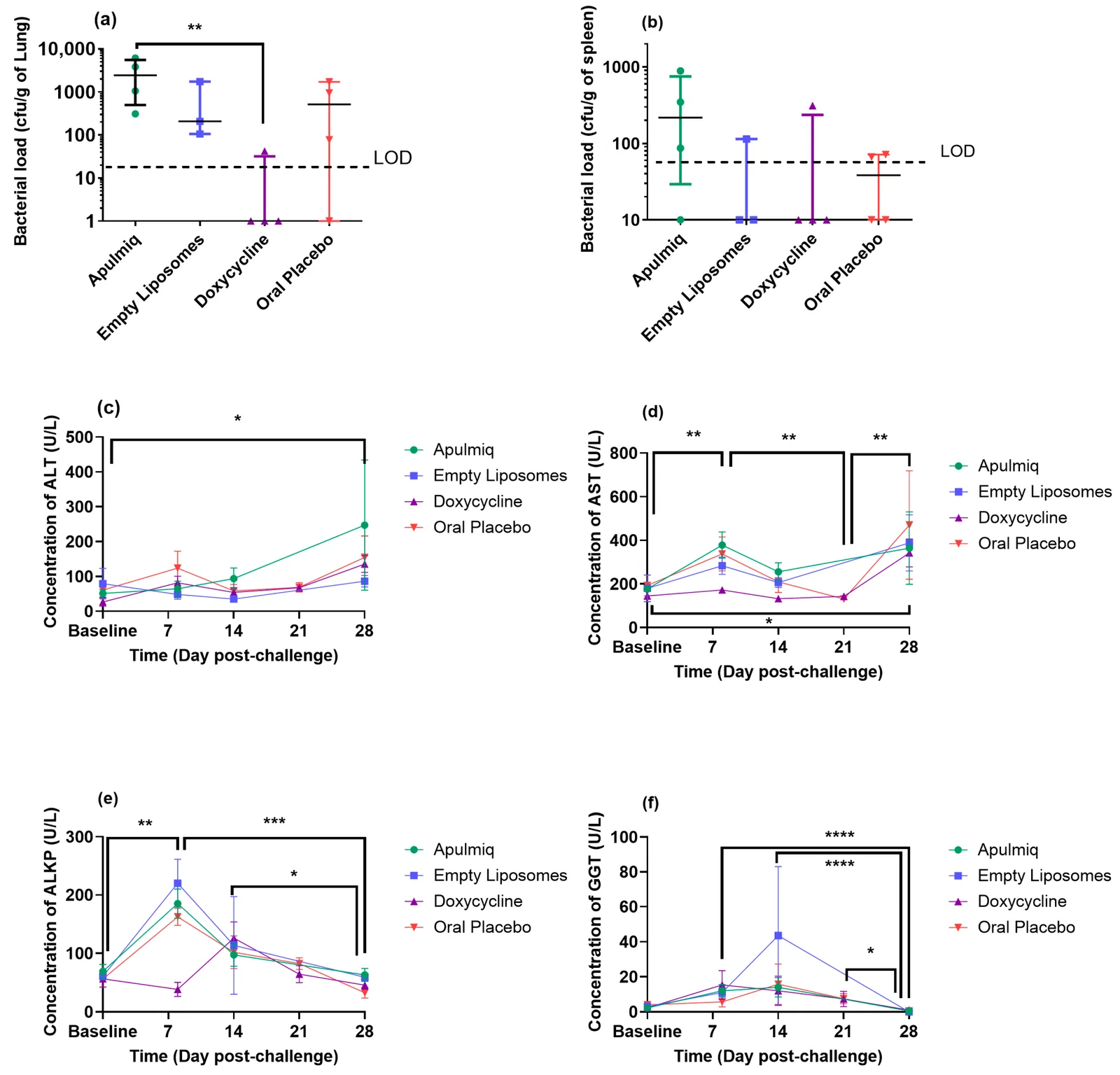
Comparison of the number of bacteria detected in the lung (**a**) or spleen (**b**) of animals at the end of the study or changes in the levels of alanine transaminase (ALT) (**c**), aspartate aminotransferase (AST) (**d**), alkaline phosphatase (ALKP) (**e**), or *γ*−glutamyl transferase (GGT) (**f**) during disease progression. Significant differences between each timepoint were determined using ANOVA (a and b) and Kruskal–Wallis (**c**–**f**) and are denoted by stars (* *p* < 0.05, ** *p* < 0.01), *** *p* < 0.001, **** *p* < 0.0001. There are 4 animals per group and the error bars represent the median with the interquartile range.

Bacteria were recovered from the lungs of at least one animal per group at the time of post-mortem (Day 28) (11 out of 15) (Figure 5a). There were significantly less bacteria in the group of animals that received doxycycline compared to Apulmiq (*p* < 0.01), with three animals completely clearing the bacteria from the lungs. A similar pattern was observed in the spleens of these animals, with 3 out of the 4 animals that received Apulmiq being colonised (Figure 5b). The animal that was euthanised on Day 8 post-challenge that received empty liposomes had high levels of bacteria in the lungs and spleen, 1 × 10^6^ cfu and 6.1 × 10^4^ cfu, respectively. No bacteria were recovered from the genitals, bone marrow, or adipose tissue of any animal on Day 28.

Levels of liver enzymes in the plasma were compared pre-challenge (baseline) to blood collected on Days 8, 14, and 21 and at the end of the study (Day 28). Levels of alanine transaminase (ALT) were significantly increased on Day 28 post-challenge (*p* < 0.05) (Figure 5c). However, there were significant increases in levels of aspartate aminotransferase (AST) and alkaline phosphatase (ALKP) on Day 7 post-challenge compared to baseline for all groups except the animals that received doxycycline (*p* < 0.01 in both cases) (Figure 5d,e). Levels declined on Days 14 and 21 for both enzymes and continued to return to within a normal range on Day 28 for ALKP. However, levels of AST significantly increased on Day 28 compared to levels on Day 21 (*p* < 0.01). In the group of animals that received empty liposomes, levels of γ-glutamyl transferase (GGT) increased on Day 8 and further increased on Day 14 before returning to normal levels on Day 28 (Figure 5f).

### 2.3. Systemic Neutrophils Were Activated Despite No Significant Increase in the Proportion of Neutrophils

**Figure 6:**
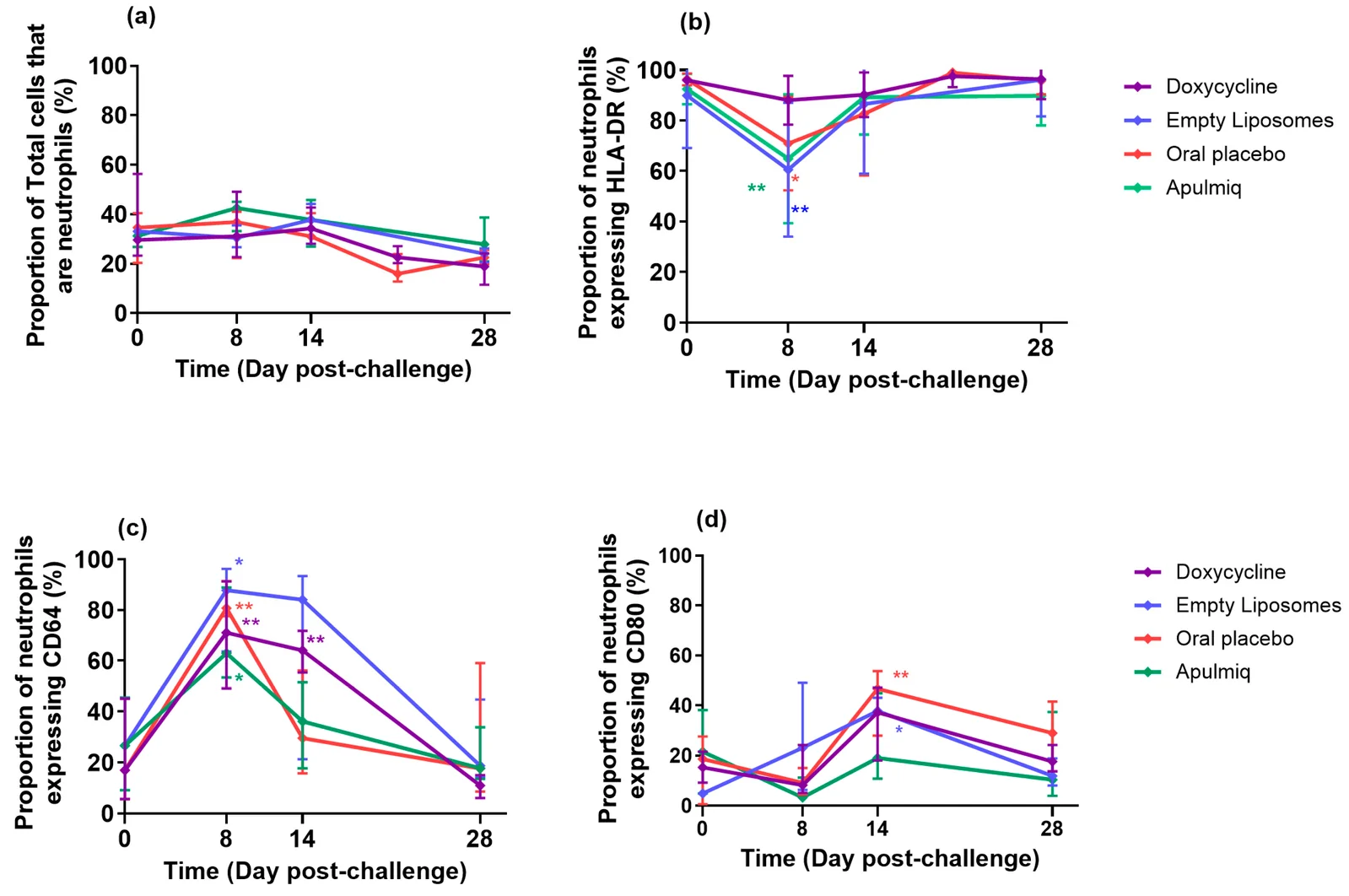
The percentage of neutrophils (**a**) and their expressions of HLA-DR (**b**), CD64 (**c**), and CD80 (**d**) in the blood of marmosets following challenge with *C. burnetii* and administration of therapy by either the oral or inhalation routes. Significant differences from the pre-challenge level (baseline) were determined by one-way ANOVA and are denoted by stars (* *p* < 0.05, ** *p* < 0.01). There are 4 animals per group and the error bars represent the median with the interquartile range.

The numbers of neutrophils in the blood did not change significantly with disease progression or alter between the different study groups (Figure 6a). However, the levels of activation markers did change throughout the disease course. The expression of HLA-DR significantly decreased in all groups on Day 8 except in the animals that received doxycycline (*p* < 0.05 placebo animals, *p* < 0.01 for Apulmiq and empty liposome groups) (Figure 6b). Reduction in this marker on marmoset neutrophils is characteristic of infection. The levels then returned to normal by Day 14 post-challenge. The levels of CD64 expression significantly increased on Day 8 for all cohorts of animals, with the greatest increase observed in animals that received Apulmiq (Figure 6c). The CD64 expression levels remained elevated for both the doxycycline and Apulmiq groups, although only statistically significantly for the doxycycline group. The levels returned to normal by Day 21 post-challenge. Increased expression of CD64 expression on neutrophils is linked to sepsis in humans. The levels of CD80 expression increased in all groups, peaking on Day 14, significantly for the empty liposome and oral placebo groups (*p* < 0.01 and *p* < 0.05, respectively) (Figure 6d). CD80 expression returned to baseline levels by Day 28. CD80 expression allows neutrophils to activate T-cells, initiating the adaptive immune response.

The animal that succumbed (empty liposome group) to infection on Day 8 was excluded from the above analysis. In this animal, the proportion of neutrophils was within the same range as the other animals, with high expression of CD54, suggesting the neutrophils were in the process of migrating to infected tissues. The expression of CD64 was relatively low and HLA-DR relatively high for an animal fighting an overwhelming acute infection.

### 2.4. A Classical Activated Macrophage Response Was Observed in the Acute Disease, Switching to an Alternative Response by the End of the Study

**Figure 7:**
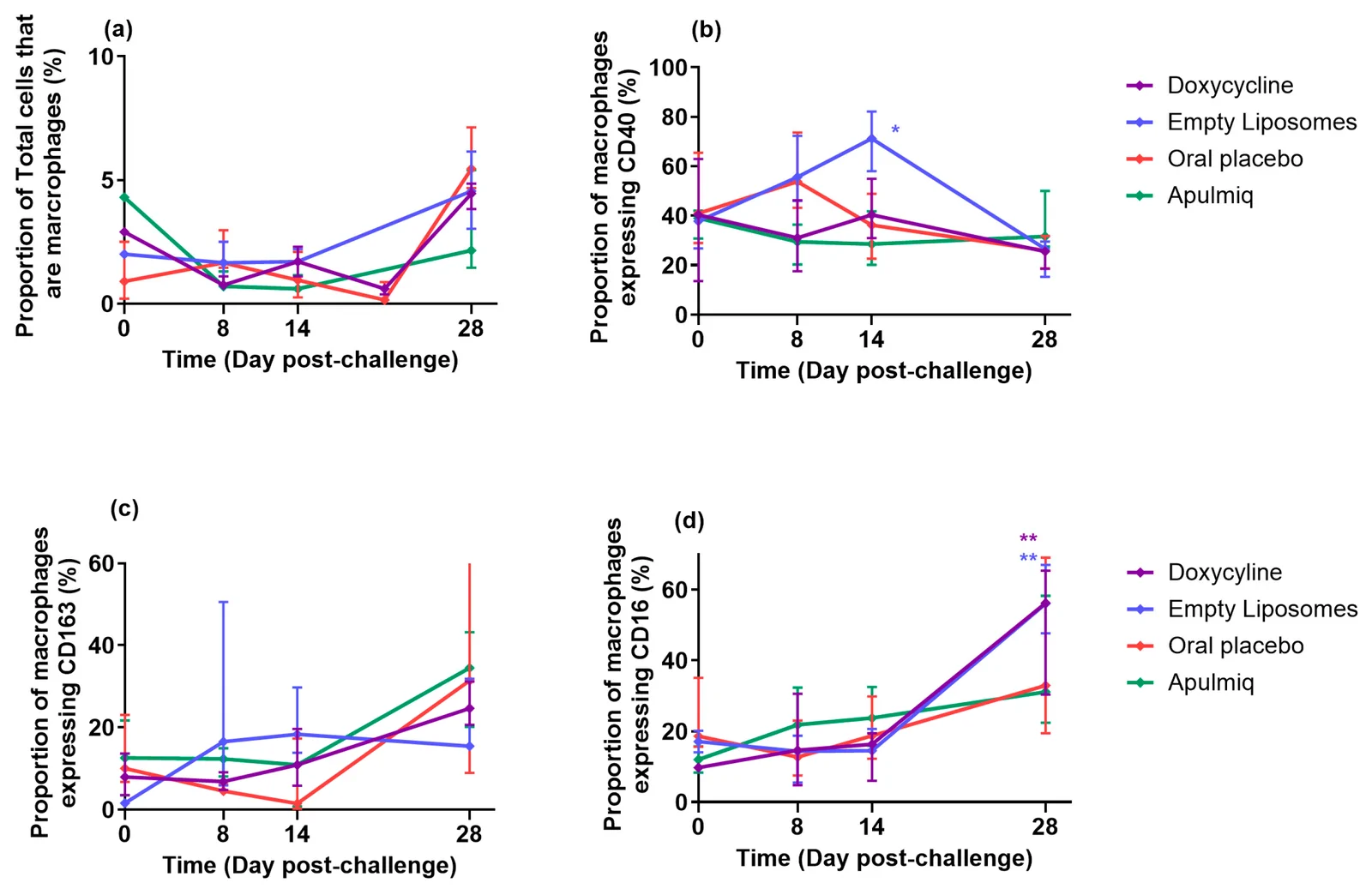
The percentage of macrophages (**a**) and their expression of CD40 (classically activated) (**b**), CD163 (alternatively activated) (**c**), and CD16 (intermediately activated) (**d**) in the blood of marmosets challenged with *C. burnetii* and administered therapy by either the oral or inhalation routes. Significant differences from the pre-challenge level (baseline) were determined by one-way ANOVA and are denoted by stars (* *p* < 0.05, ** *p* < 0.01). There are 4 animals per group and the error bars represent the median with the interquartile range.

The numbers of monocytes in the blood decreased at Day 8 post-challenge and remained low prior to increasing by Day 28 post-challenge (Figure 7a). The expression of CD40 (classical activation marker) increased in the oral placebo or the empty liposome groups when measured at Day 8 post-challenge, and continued to increase in the animals that received empty liposomes (Figure 7b). The levels returned to normal in all groups by Day 28 post-challenge. The expression of CD163 (a marker of alternative activation marker) and CD16 increased between Day 14 and Day 28 in all cohorts of animals, peaking at Day 28 post-challenge, except for the CD163 expression in animals that received empty liposomes (Figure 7c,d).

The animal that succumbed (empty liposome group) to infection on Day 8 was excluded from the above analysis. In this animal, the proportion of monocytes was at the higher level of the group range, with high expression of both CD80 and CD40 suggesting activation of the macrophages. However, the expression of CD163 was four-fold higher than any other animal (61% vs. 15%). High expression of CD163 implies that the macrophages were activated through the alternative pathway, a condition that is known to favour the growth of *C. burnetii* within the macrophages.

**Figure 8:**
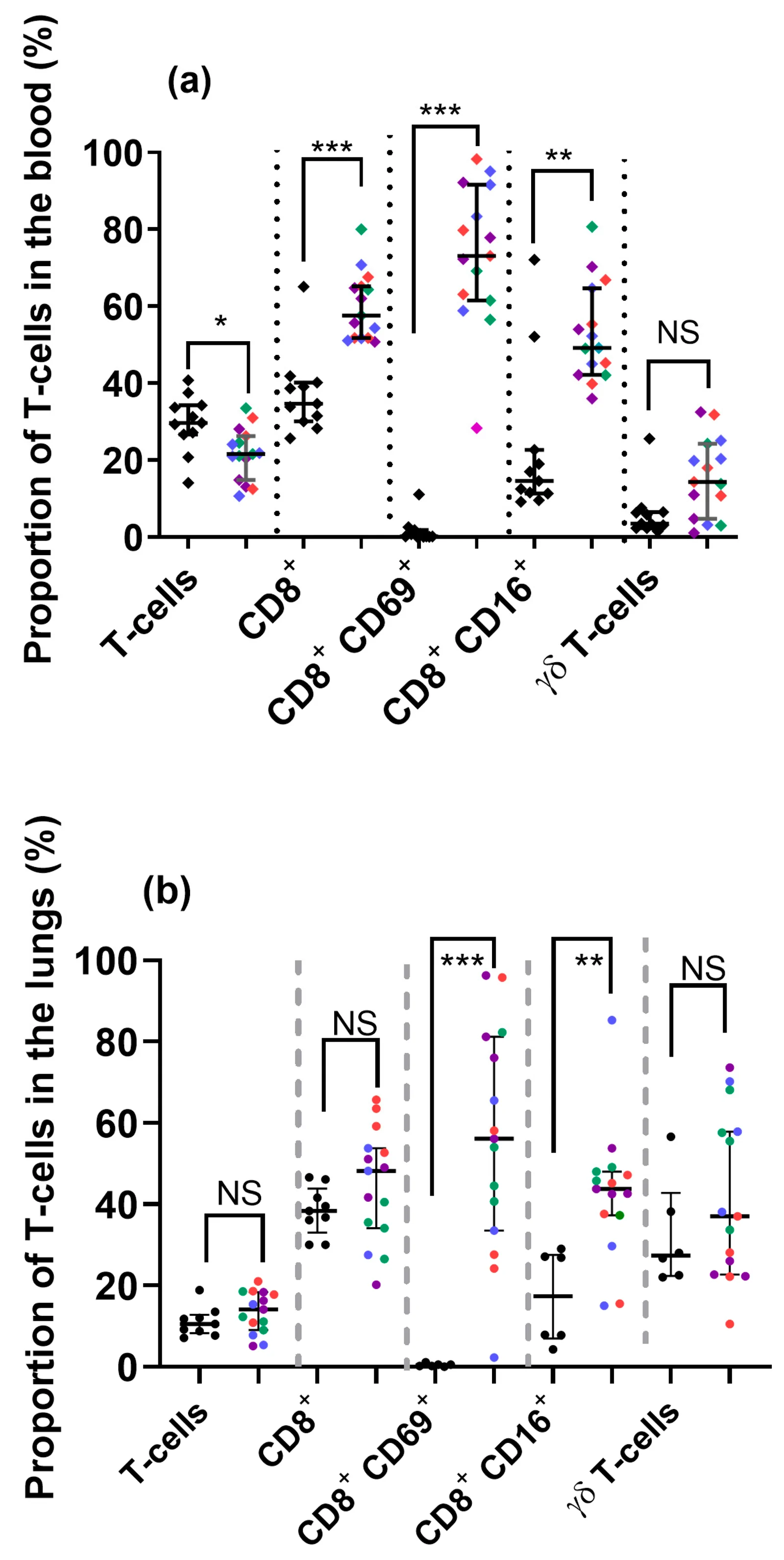
The percentage and expression of markers on T-cells in the blood (**a**) or lungs (**b**) of marmosets challenged with *C. burnetii* and administered therapy by either the oral or inhalation routes at the end of the study (Day 28). Significant differences from the pre-challenge level (baseline—black dots) were determined by one-way ANOVA and are denoted by stars (* *p* < 0.05, ** *p* < 0.01, *** *p* < 0.001, NS is not-significant). There are 4 animals per group and the error bars represent the median with the interquartile range. Black dots—baseline vlaues, green dots—Apulmiq group, blue dots—empty liposome group, purple dots—doxycycline group, and red dots—oral placebo group.

The number of CD3^+^ T-cells in the blood remained relatively constant during the study; however, they were highly activated by Day 28 (Figure 8a). There was a significant increase in the CD3^+^CD8^+^ population (*p* < 0.001), the CD3^+^CD8^+^CD69^+^ population (*p* < 0.001), and the CD3^+^CD8^+^CD16^+^ (*p* < 0.01) population. Levels of γδ T-cells may have also increased; however, not to a significantly determinable level. There was no difference associated with any group.

### 2.5. Neutrophil and Macrophage Activity in the Lungs Continued for the Duration of the Study to Promote Bacterial Clearance

There was no difference in the quantity of neutrophils in the lungs between any group of animals on Day 28. However, the animal that was euthanised on Day 8 had a high level of neutrophils (54.7%), which is consistent with pneumonic disease. These neutrophils were CD16^-^, indicating an influx of relatively immature neutrophils. Correspondingly the expressions of CD64, CD54, CD66b, and CD80 on neutrophils was low, further suggesting neutrophil immaturity in the lungs of this animal. In comparison, the surviving animals had higher levels of CD64, CD54, and CD80 and lower HLA-DR and CD16 expression than naïve animals, suggesting on going activity to ensure clearance. Similarly, there was no difference between the groups in terms of macrophage numbers and expression; however, all macrophages had a greater CD80 expression and a lower CD16. This suggests classically (aggressively) activated macrophages that were involved in the final clearance of bacteria from the lung tissue. Animals that received empty liposomes generally expressed high levels of CD16 and low levels of CD40, which is correlated to the higher bacterial counts in the lungs of these animals. One exception to this was a low level of CD80 expression on lung macrophages in the animal that was euthanised on Day 8. There was no statistically significant difference in the number of lymphocyte cells in terms of the proportions of B cells, T cells, CD8^+^ T cells, or γδ T-cells on Day 28 post-challenge compared to those found in naïve animals (Figure 8b). However, the expressions of activation markers on the T cells, especially CD16 and CD69, were significantly increased. This occured regardless of antibiotic group.

### 2.6. IFN-γ Was the Predominant Cytokine Response to Infection

The levels of a number of cytokines (IFN-γ, TNF-α, IL-2, IL-17, IL-6, IL-1β, MCP-1 (CCL2), and RANTES (CCL5)) were assessed in the plasma of animals collected on Days 8, 14, and 28. The cytokine level was also assessed in lung and spleen homogenates on Day 28. No IL-17 or IL-2 was detected in any sample including samples obtained from the animal that succumbed to disease. The levels of IFN-γ could be related to the disease. The highest levels of IFN-γ were observed in the plasma of the control cohorts of animals (oral placebo and empty liposomes) on Day 8 post-challenge. No IFN-γ was detected on Day 8 in animals that received doxycycline, but it was detected in 3 out of 4 animals on Day 14. Detectable levels of IFN-γ were associated with the febrile response and bacteraemia; 90% of animals that were febrile had measurable IFN-γ (18/20) and 90% of those not in fever had no detectable IFN-γ (10/11). A total of 80% of animals with detectable IFN-γ also had viable bacteria (14/17). However, the concentration of IFN-γ was not significantly linked to the quantity of viable bacteria. One animal that received doxycycline surprisingly had low levels of IL-6 in the plasma on Day 8 (500 pg/mL). Another animal that received doxycycline and one animal that received Apulmiq had 100 pg/mL of TNF-α in the plasma on Day 14. The cytokine expression for all other animals was below the limit of detection.

The animal that succumbed to infection had high levels of IFN-γ and IL-6 in the plasma, and high levels of IFN-γ, TNF-α, IL-6, MCP-1, and RANTES in the lung sample. Three other animals had IFN-γ in their lung samples (Day 28), two that received empty liposomes (3.8 and 13.6 pg/mL, respectively) and one that received Apulmiq (12.5 pg/mL). All these animals also had detectable bacteria in the lungs.

### 2.7. Treated and Untreated Animals Developed a Memory Response Following Challenge

**Figure 9:**
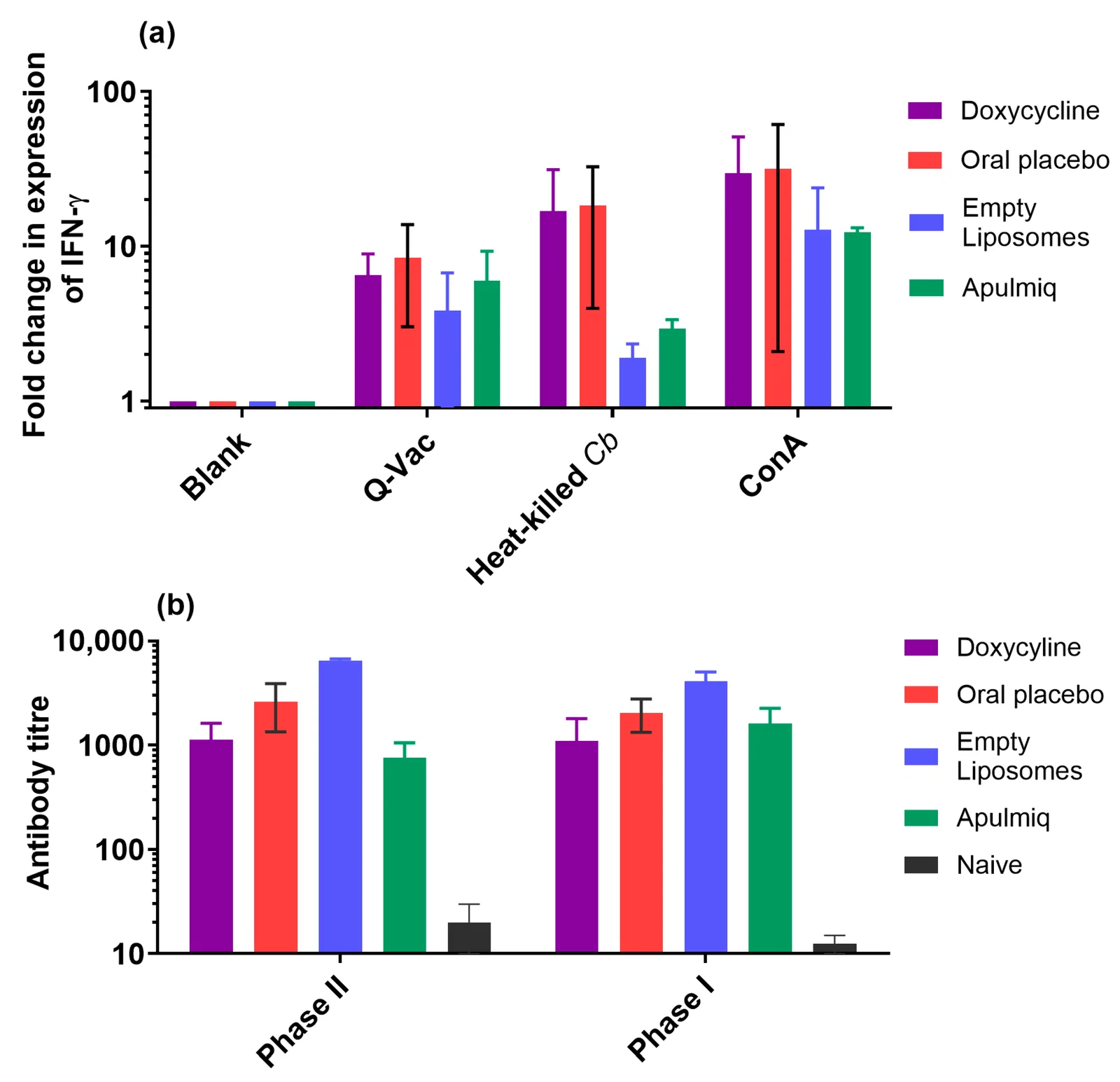
Memory response following spleen stimulation (IFN-γ) (**a**) or antibody in the plasma at the end of the study (**b**) in marmosets euthanised on Day 28 following challenge with *C. burnetii* and administered therapy by either the inhalation or oral routes. Blank is the unstimulated control, heat-killed *C. burnetii* is 1 × 10^7^ cfu/mL of heat-killed *C. burnetii*, Q-vac is the vaccine (1 in 10), and ConA is the 2.5 μg/mL positive control. There are 4 animals per group and the error bars represent the standard deviation.

Spleen homogenates from animals euthanised on Day 28 post-challenge were stimulated with either heat-killed *C. burnetii* (HK Cox) at a concentration of 1 × 10^7^ per mL or a 1:10 dilution of the Q-Vax vaccine. Levels of IFN-γ above the background levels indicated an antigen-specific cell-mediated immune response (CMI) (Figure 9a). CMI was observed in all groups, with a higher response associated with the heat-killed bacteria stimulation. Higher stimulation was observed in animals that received either oral doxycycline or placebo compared to those that received the inhalational Apulmiq or empty liposomes, although there was no statistical significance.

Antibody titres, in plasma obtained on Day 28, to heat-killed Phase I and Phase II bacteria were measured (Figure 9b). Phase I and Phase II IgG antibodies were detected in all animals, with the exception of one of the animals that received Apulmiq whose levels were borderline. However, animals that did not receive any antibiotic had slightly higher levels, which was not statistically significant.

### 2.8. Statistical Analysis of the Data Indicates Fever and Bacteraemia Correlate Strongly with Protection

**Figure 10:**
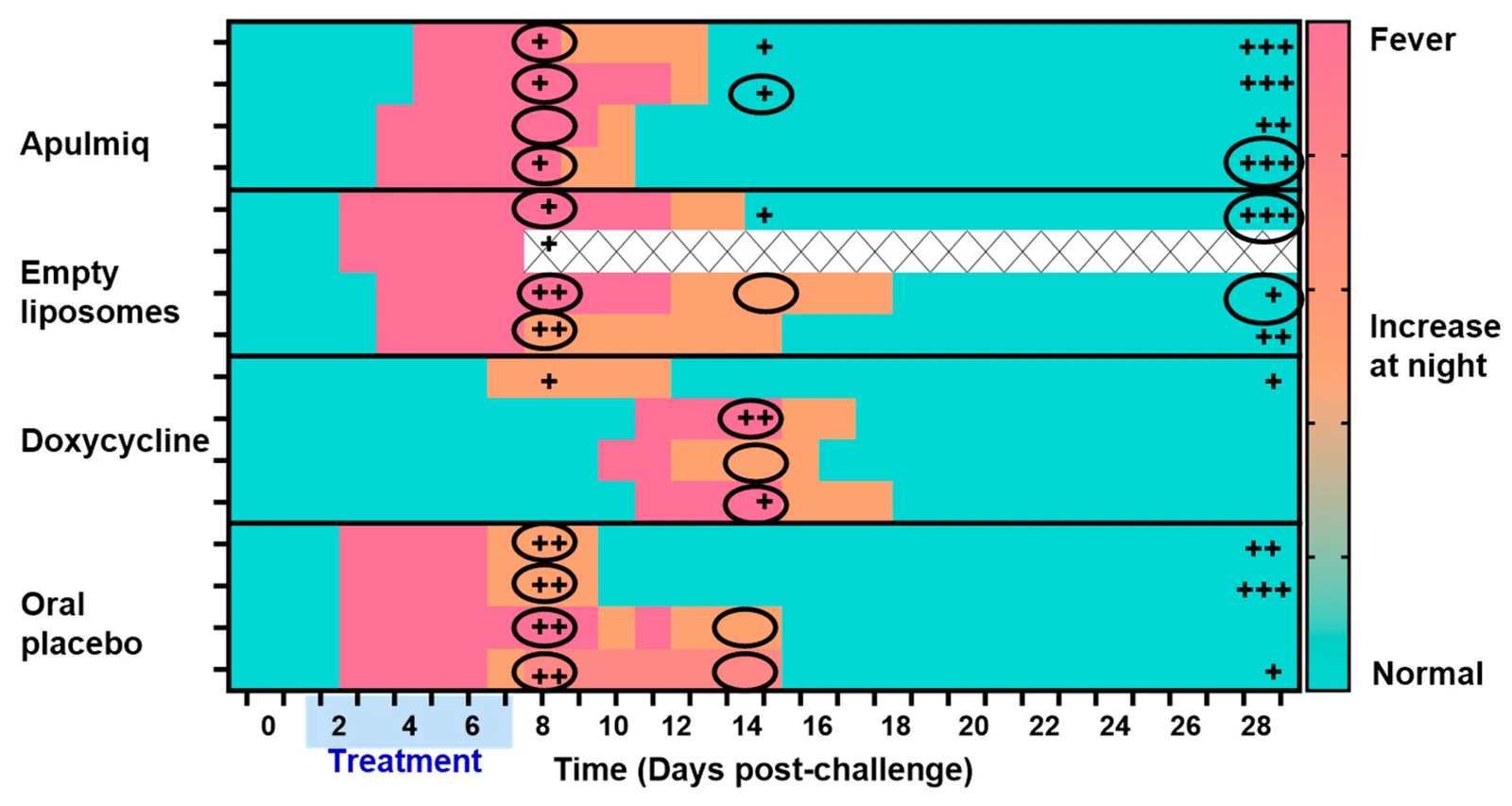
Heat map showing the temperature profile of marmosets challenged with *C. burnetii* and administered doxycycline or placebo by the oral route or Apulmiq or empty liposomes by the inhalation route once a day for 7 days. Bacterial load is represented by + (20–100 cfu), ++ (100–500 cfu), or +++ (500 to 6200) in the blood on Days 8 and 14 or lung on Day 28. Circles indicate the detection of IFN-γ. The white hatched area indicates there was no data for this animal as they had been euthanised.

A pictorial representation of the course of the disease in all animals is shown in Figure 10. This highlights the relationship between the presence of fever and bacteraemia and systemic IFN-γ.

**Figure 11:**
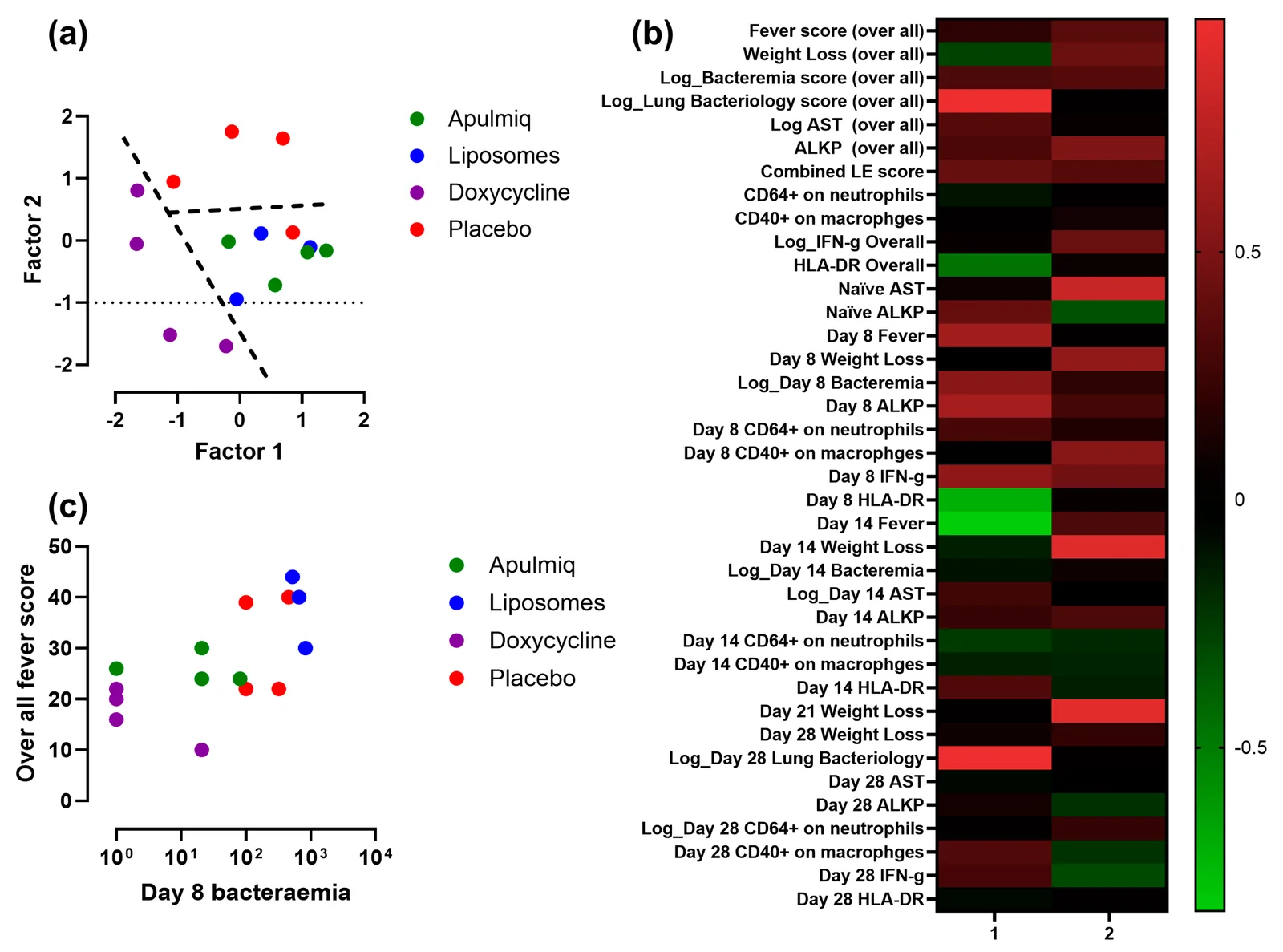
Statistical analysis of the data generated in marmosets challenged with *C. burnetii* and administered doxycycline or placebo by the oral route or Apulmiq or empty liposomes by the inhalation route. (**a**) Factor analysis showing each marmoset in relation to the first two factors of the analysis. Lines have been added to illustrate separation between groups. (**b**) The relative contribution (−1 negative to +1 positive correlation) of the 48 parameters measured in marmosets to each of the first two components of the PCA. (**c**) The relationship between two parameters indicated to be predictors of doxycycline administration by multinomial stepwise regression (overall fever score and day 8 bacteraemia level). Overall fever score was the cummulative score over the study period based on whether the animal had a fever (4 units) or whether they only had a raised temperature at night (2 units), and was used as a numerical way of comparing animals.

To explore this more broadly, multivaraite analysis was performed in a non-subjective way to interpret all the parameters assessed for each animal. The dataset comprised 48 variables, describing 15 of the marmosets (one animal was not considered due to the limited data available as this animal succumbed to disease during the course of the study). Standard regression imputation was required for only 10 of the 570 data points, due to missing data. Factor analysis was used to understand the relationship between the parameters in the context of other factors that delimit the individuals (Figure 11). However, this expolartory analysis needs to be treated with caution due to the small number of animals as there is a potential for the data to be overfitted. The analysis indicated 26% of the total variance across this dataset could be accounted for by making a composite factor of measurements that correlated well together. A second factor was able to account for approximately 16% of the total variance. Each marmoset was considered in terms of these two factors (Figure 11a). These two factors provided a good degree of separation for both the doxycycline-treated group, which were low in both components 1 and 2. There seemed to be a negative correlation between these components, in that doxycycline-treated animals seemed to be either very low in factor 1 and central in factor 2 or low in both. This suggests that PEP with doxycycline had a meaningful effect on the biology of the animals during infection. This analysis also provided a good level of separation for the placebo group, which was high in factor 1. Therefore, these factors can distinguish between untreated animals and those with the best outcome. The parameters that were strong contributors to this relationship (factor 1) included HLDR expression, fever and bacteriology (factor 1), and weight loss (factor 2) (Figure 11b).

Stepwise multiple regression was performed to iteratively select only the best independent predictors for doxycycline administration. This method ignores explanatory measures that correlate with variables already added to the model and move preferentially to the next-best predictor, which does not correlate with the previously determined measurement. Through this iterative process, the potential for false positives within the individual test is reduced and improves collinearity. Two predictive, independent parameters were identified, day 8 bacteraemia (*p* < 0.001) and overall fever score (*p* = 0.034) (Figure 11c). These were also important in factor 1 of the PCA, indicating their strong impact in the relationship between untreated animals and those that received doxycycline. IFN-γ was identified as important in the PCA analysis but the levels of IFN-γ were strongly correlated to bacteraemia, and so it was “rejected” in the multiple regression analysis in favour of day 8 bacteraemia. Interestingly, the data for the animals that were treated with Apulmiq in the overall fever/bacteraemia plot lies between the doxycycline and untreated animals. This suggests a moderate level of protection from this antibiotic.

## 3. Discussion

The aim of this work was to compare the efficacy of inhalational ciprofloxacin (Apulmiq) with the standard of care antibiotic for Q fever in an animal model closely related to humans. Acute Q fever is treated with twice-daily administration of doxycycline for 14 days at a dosage of 100 mg [4]. This reduces the fever within 2–3 days of administration. However, relapse can occur following cessation of antibiotic administration and chronic disease has been associated with poor chemotherapeutic management [6,18]. Oral ciprofloxacin is not recommended for treating acute Q fever but has been successful in the treatment of chronic Q fever complications, specifically endocarditis [19,20]. The use of liposomal encapsulated antibiotic formulations will change the pharmacokinetics and pharmacodynamics, facilitate slow release of the drug, and provide high local drug concentrations that may reduce the number of doses required [7,21]. In addition, delivery of the antibiotic to the lung targets the site of infection of respiratory disease and can improve patient outcome [8]. Inhalation delivery of liposome-encapsulated ciprofloxacin demonstrated superior protection to systemic ciprofloxacin when delivered intra-nasally in a murine model of Q fever [11]. Reduced weight loss and clinical signs, such as reduced splenomegaly and bacterial colonisation of the lungs and spleens, were observed. The use of an inhaled formulation of doxycycline was not available as a comparator and was outside the scope of this study.

A marmoset model of Q fever was developed that exhibits many of the key features of human Q fever [15]. This was used to compare the efficacy of the two antibiotics, doxycycline and Apulmiq. Apulmiq (formerly known as Pulmaquin) is a 1:1 volume-to-volume mixture of liposome-encapsulated ciprofloxacin and a non-liposomal solution of 20 mg/mL of free ciprofloxacin expressed as hydrochloride salt (or 18 mg/mL of ciprofloxacin base) [8]. Dosing regimens for Apulmiq to deliver human-equivalent doses to marmosets were previously established at Dstl [14]. Inhalation delivery of Apulmiq for 30 min was sufficient to deliver 0.8 mg/kg to the animal. The human-equivalent dose of doxycycline was determined in this study. In order to deliver an appropriate dose to marmosets in the efficacy study, the initial starting dose of 10 mg/kg was based on the human dosage and scaled in accordance with the FDA Guidance [22]. A single dose of oral doxycycline was administered to the animals and the pharmacokinetics were determined. The time to maximum concentration (T_max_) of 1.1 h was comparable to the low end of the range observed in humans administered the same dose of between 1 and 4 h [23]. However, the maximum concentration (C_max_) and AUC (3.8 mg/L and 64.9 h.mg/L, respectively) were higher than that observed in humans (C_max_ of 1.7 to 2 mg/L, AUC of 12.7 to 40.1 h.mg/L). Compartmental modelling was performed to determine an appropriate dose to reach the target therapeutic AUC range of 13 to 40 h.mg/L [16]. Modelling predicted that a regimen of 4 mg/kg of doxycycline administered once daily would result in an AUC of 20.1 h.mg/L. This also aligns well with the AUC in mice that was effective for Q fever [11]. Therefore, this regimen was assessed in a multiple dosing study. However, compartment modelling was not able to fit the data generated following multiple doses at both Day 1 and Day 5. In addition, the observed accumulation index was approximately twice the predicted accumulation index. Both of these findings are suggestive of a non-linear relationship. Atypical non-linear protein binding has previously been reported for doxycycline in humans [24]. The precise mechanism underlying the non-linearity observed in the present study remains unclear. In addition to altered protein binding, other factors may contribute, including species-specific differences in tissue distribution, saturation of transport processes involved in absorption or biliary excretion, changes in enterohepatic recirculation, or time-dependent alterations in drug disposition following repeated dosing. The relative contribution of these mechanisms could not be determined from the current data. Nevertheless, the discrepancy between the predicted and observed accumulation demonstrates that pharmacokinetic parameters derived from single-dose studies may not accurately predict steady-state exposure for doxycycline in this model. This highlights the need for caution when extrapolating doxycycline exposure from single-dose pharmacokinetic studies to multiple-dose efficacy studies. Second, it suggests that exposure may increase disproportionately with repeated dosing, potentially affecting both therapeutic efficacy and the risk of adverse effects. While the clinical significance of the reported concentration-dependent protein binding has not been established in either human studies or animal models, the present results indicate that non-linear processes should be considered when selecting doxycycline dosing regimens for marmosets.

Dose selection was guided primarily by the experimental pharmacokinetic data, although the daily doses of 4 and 10 mg/kg were unlikely to achieve the desired AUC range. A dose of 6 mg/kg once daily was therefore selected as a pragmatic compromise. This aimed to increase exposure above the single-dose AUC of 8.4 h·mg/L observed following 4 mg/kg while avoiding the substantially higher exposure associated with the 10 mg/kg dose. However, this intermediate dose was not empirically assessed in the pharmacokinetic studies prior to efficacy evaluation, and future work should characterise its exposure profile directly and further investigate the mechanisms responsible for the observed non-linear pharmacokinetics.

Marmosets were successfully challenged with 3.2 × 10^6^ ± 1.1 × 10^6^ cfu of *C. burnetii* by the aerosol route. Administration of antibiotic was initiated 24 h post-challenge, at which time there was no overt clinical signs or febrile response in the animals. The administration of antibiotic continued for 7 days, although this is less than the recommended duration of 14 days [4]. The suboptimal treatment conditions used in this study were designed to allow a more sensitive assessment of potential differences between the groups. Administration of either antibiotic (doxycycline or Apulmiq) reduced the disease outcome to some extent. An increase to the time of fever was observed as well as a reduction and/or delay in weight loss, less severe pathological features in the lungs, and reduced bacterial colonisation compared to the controls. Doxycycline-treated marmosets generally fared better than those given Apulmiq, and weight loss and fever were delayed until the antibiotic dosing had cessed. This delay in weight loss following early administration of doxycycline was also observed in the murine model of Q fever [11,17]. Apulmiq therapy delayed the onset of fever and reduced the level of bacteraemia during the first 8 days of the disease. This indicates that the inhaled antibiotic is able to initially control the disease and reduce bacterial dissemination and potentially replication. However, as high levels of bacteria were present in the lungs on Day 28, this did not continue throughout the course of disease. Extended dosing with Apulmiq may help to control the bacterial numbers further, and may delay the onset of treatment. Early initiation of antibiotic, as undertaken in this study, may actually be detrimental, as low concentrations of bacteria may not trigger a robust host inflammatory response to aid in bacterial clearance. Indeed, murine data [17] and historic human data [25] suggest that prophylactic treatment of Q-fever can be detrimental to outcome.

Doxycycline is a bacteriostatic antibiotic that slows the replication and spread of the bacteria. In optimal treatment regimens, this would allow the development of an appropriate adaptive immunological response that would then eradicate the bacteria. The timing of doxycycline administration may be key in supporting the immune response, as described above. Later initiation of antibiotic therapy may also be detrimental as the bacteria numbers may be above threshold levels for doxycycline, and thus the bacteria may be “pushed” into unusual niches, such as the pericardium, resulting in chronic Q fever with endocarditis complications. The data in mice and marmosets suggests that antibiotic administration improves disease outcome, especially when assisted by a primed classical immune response to *C. burnetii*. This is clearly demonstrated in the marmoset model and it would be intriguing to explore delayed therapy in the model.

The immunological changes in the cell proportions and their phenotypes were in line with expected/published work [15,18,26,27,28]. There was a limited but measurable response from the neutrophils. Neutrophils are the first responders to bacterial infections, but as Q fever is not a severe disease, there was not a large change in proportions. The reduction in HLA-DR expression on these cells appears to be a marmoset-specific response to infection [29]. There was also an increase in neutrophil CD64 expression, which has been increasingly used as a biomarker to diagnose infection and sepsis in humans [27]. The macrophages are known to be crucial in the control of Q fever. *C. burnetii* is able to replicate in alternatively activated macrophages, but killed by classically activated ones [28]. During the course of disease, the circulating monocytes expressed a more classical (aggressive) phenotype; once the infection was conquered, they developed a more intermediate/alternative phenotype (associated with repair and recovery). The classical activation status of the monocyte/macrophages is largely controlled by the levels of circulating IFN-γ [26,30]. During the disease, especially when marmosets were bacteraemic and in fever, IFN-γ was detectable. This presumably played a critical role in ensuring the macrophages remained in their aggressive state, controlling the *C. burnetii* growth [31]. IFN-γ was the only cytokine found that showed any relationship to disease, which has been observed in both mouse and marmoset studies at Dstl and reported in humans [26,30].

Towards the end of the infection, there was evidence of T-cell activation, in line with a developing adaptive immunity that will prevent the animal from further infection. Not only were the T cells phenotypically active, but the re-call/re-stimulation assay showed that this response was directed against *C. burnetii*. Finally, along with a good cell-mediated (T-cell) response, there was also evidence of a building antibody response, suggesting these animals followed the path of human disease [32].

A unique immunological response was observed in two animals: the one animal that was euthanised on Day 8 and one animal that received Apulmiq. The animal that was euthanised had a pronounced pneumonia that was not previously observed in animals challenged with *C. burnetii.* This was evidenced by a build-up of neutrophils in the lungs, which was confirmed by histology. Despite high levels of circulating IFN-γ, the circulating monocytes were more phenotypically alternative, allowing the uncontrolled replication of *C. burnetii*. The animal also had measurable levels of IL-6, which is associated with more severe disease in humans [33].

One animal that received Apulmiq, and never had bacteraemia, had very limited IFN-γ production, had no detectable CMI response, and a low antibody response. The neutrophils in the animal’s blood on Day 8 appeared unaffected, although there was a limited number of viable bacteria found in the lungs on Day 28 (311 cfu/g). Despite developing fever, it seems that this animal controlled the bacterial numbers well enough to not trigger a proper immune response. Seroconversion without symptoms is well documented in human cases [34], whether it can happen the other way around via signs without conversion and protection, is not known. It may be that this animal would have gone on to relapse and develop some long-term *C. burnetii-*associated condition. This clearly demonstrates the phenomenon described above where initiation of treatment too early may be detrimental and not allow development of an effective immune response.

A fully developing immune response was observed in all but one animal and is likely to be because the antibiotic regimens allowed the animals to develop some level of disease. The disease appeared most severe in those treated with the empty liposomes. The reason for this is unclear, as the liposomes were expected to increase the number and classical (aggressive) activation of the macrophages. In the blood, the circulating monocytes had higher levels of CD40 or CD80 expression, indicating that they were activated, but this coincided with higher counts in the blood. The status of the macrophages in the lungs early in the disease process is unknown, but there may have been immediate recruitment to the lungs, resulting in a favourable environment for *C. burnetii*.

Finally, this study helped validate the important metrics in marmosets necessary to assess medical countermeasures. Q fever is typically a non-lethal model and relies on well-characterised parameters other than survival. Previously, our group hypothesised that fever would be the primary readout in the marmoset model, and weight loss, bacteraemia/bacterial load in the lungs, liver enzymes, expression of CD64 on neutrophils, and CD40 on macrophages would be useful secondary readouts [15]. The protection afforded by doxycycline, and to a lesser extent Apulmiq, was clearly demonstrated with defervescence as the primary indicator. Differences in the treated and untreated groups were also observed with the secondary parameters of weight loss, bacteraemia, and increase in AST and ALKP on Day 8 and bacterial load in the lungs on Day 28. Reduced bacteraemia on Day 8 (i.e., at the cessation of antibiotic) was the only secondary readout to indicate a benefit of Apulmiq administration. Fever and Day 8 bacteraemia were highlighted by the multivariate regression analysis as the key parameters predicting antibiotic groups and therefore a successful outcome. Changes in the immunological markers, CD40 on macrophages and CD64 on neutrophils, were useful retrospective indicators that could be used to inform why antibiotics succeeded or failed but do need to be considered in the context of other readouts. For example, on Day 8, levels of CD64 were increased in all groups, indicating infection, but by day 14, the levels had decreased in the control groups, as clearance and recovery were in progress. In the treated groups the levels remained high on Day 14, suggesting the antibiotic was holding back the infection and, consequently, clearance at this point occurred more slowly. For CD40 expression, levels in antibiotic-treated animals remained low, with a slight peak in the doxycycline animals on Day 14, which again indicates either delayed or non-immunological control of disease.

## 4. Materials and Methods

### 4.1. Animals

Healthy, sexually mature common marmosets (*Callithrix jacchus*) were obtained from the Dstl Porton Down breeding colony and housed in female and vasectomised male pairs. Animals were aged between 1.4 to 5 years old and weighed between 386 and 538 g at the time of study. Diet and a variety of food were provided daily and irradiated water was provided ad libitum. Animal rooms were illuminated with fluorescent lights and maintained on either a 12 h light/dark cycle (non-containment) or 14/10 h light/dark cycle (containment). Temperature was maintained at between 23 and 28 °C and RH maintained between 40 and 70%. Environmental enrichment included deep litter foraging, Tupperware boxes, and various sized and textured perches. Prior to use in challenge studies, all animals were surgically implanted intra-peritoneally (i.p.), under general anaesthesia (ketamine/medetomidine/isofluorane), with a Remo 201 device to record core body temperature (Tc) remotely (EMMS Bordon, Hampshire, UK). Prophylactic pain relief of 0.2 mg/Kg of meloxicam and 0.005 mg/Kg of buprenorphine was administered. Data were analysed using the eDacq software to provide real-time and recordable Tc (EMMS, Bordon, Hampshire, UK). At least 7 days prior to the day of challenge, blood was collected from all animals to assess baseline levels of immunological markers. Animals were anesthetised with 5 mg of ketamine hydrochloride and a 23-gauge needle was attached to a 5 mL syringe and up to 2.5 mL of blood was collected from the femoral vein. Blood was also collected post-challenge at the onset of fever, and on Day 8, Day 14, and Day 21 using a cocktail of fentanyl (0.01 mg/kg), medetomidine (0.06 mg/kg), and midazolam (0.5 mg/kg) (FMM) delivered intra-muscularly [35]. Following the challenge, animals were observed at least three times a day, at eight-hour intervals. Q fever is generally a non-lethal disease, so there was no pre-determined humane endpoint for this study. A detailed unregistered study protocol was written prior to the study.

### 4.2. Determination of the Pharmacokinetics of Doxycycline in the Common Marmoset

Two studies were performed, a single dose study and a multi-dose study, with each consisting of 6 naïve, adult, common marmosets with equal numbers of males and females, with the individual animal as the experimental unit. Following an acclimation period of two weeks, the first 6 animals were administered 10 mg/kg of doxycycline, orally via a syringe. Doxycycline was prepared in a vehicle of 2 mL of banana-flavoured Nesquik^®^ powder dissolved in water. This was based on a human dosage of 100 mg for Q fever and scaled in accordance with the FDA Guidance document [22]. Blood was collected from each animal on four occasions in a sparse random design using a randomisation table. No blinding occurred during this study. Blood was withdrawn from three animals at 0.5, 1, 2, 4, 6, 8, 12, and 24 h post-dosing and collected into a lithium heparin tube, prior to mixing thoroughly. The blood was then centrifuged for 5 min at 13,000 rpm. Plasma was collected into a labelled, sterile Eppendorf tube and stored at −70 °C until analysis. Concentrations of doxycycline were determined using Liquid Chromatography–Tandem Mass Spectrometry (LC-MS) with the collection timepoint blinded to the analyst. All modelling was performed using the Phoenix WinNonLin (Pharsight v 6.1) software. Non-compartmentally modelled determined parameters such as half-life (t_1/2_), Area under the Curve (*AUC*), and maximum concentration of drug (C_max_), etc. Nonparametric superposition modelling was performed to assess the effect of changing the initial dose and the frequency of dosing. Compartmental modelling was also performed to assess the effect of changing the initial dose and the frequency of dosing. Based on this analysis, the second cohort of six animals was administered 4 mg/kg of doxycycline once a day for five days, and blood was collected after Dose 1 (Day 1) and Dose 5 (Day 5) in sparse random design, as described above. In this study, blood was collected from 4 animals at 0.5, 1, 2, 6, 12, and 24 h post-dosing. The rate of accumulation (*R_ac_*) from Day 1 to Day 2 was determined using Equation (1):

(1) $$R_{ac}=\frac{1}{\left(1-e^{-k\cdot \tau }\right)}$$

where *k* is the elimination rate constant and *τ* is the dosing interval.

The accumulation index was determined using Equation (2):

(2) $$Accumulation\;index=\frac{{AUC}_{day\;5}}{{AUC}_{0-\tau }}$$

### 4.3. To Assess the Efficacy of Apulmiq and Doxycycline Against Q Fever

Sixteen naïve, adult, common marmosets (equal numbers of males and females) were challenged with *Coxiella burnetii* Nine Mile Phase I by the inhalation route. Twenty-four hours post-challenge, cohorts of four animals were administered either an antibiotic (6 mg/kg of doxycycline administered orally via a syringe or Apulmiq by the inhalation route) or a placebo (vehicle orally via a syringe or empty liposomes by the inhalation route). This continued once daily for 7 days. Animals were randomised into cohorts using a randomisation list and no study personnel were blinded to the study group with the individual animal as the experimental unit. Animals were observed for 28 days and core body temperature was recorded by telemetry from a surgically implanted device (Remo 210, EMMS, Bordon, UK). Animals were weighed daily and blood collected on days 8, 14, and 21 and at post-mortem for bacteriological and immunological analysis.

### 4.4. Bacterial Strain and Culture

*C. burnetii* Nine Mile Phase I (NMI; RSA493) was cultured axenically in 100 mL ACCM2 broth (10 flasks in total, from a frozen stock, in a vented conical flask within a sealed container using a GENbox microaer gas generator (BioMeriuex, Basingstoke, UK). The flasks were incubated at 37 °C at 75 rpm for 6 days. The bacteria were pelleted by centrifugation (8000× *g* for 20 min on two occasions, initially as a large volume and then a smaller volume, combining multiple centrifuge pots to generate a concentrated pellet) and stored frozen at −80 °C in phosphate-buffered saline (PBS) until required. On the day of challenge, a frozen aliquot was removed and diluted to the required concentration in PBS.

For enumeration of bacteria, viable counts were performed using ACCM-2 solid media (for challenge counts and tissue burden). Viable counts were performed by ten-fold serial dilution by plating multiple 100 µL aliquots onto ACCM-2 solid media, which were incubated statically for 14 days at 37 °C with the atmosphere adjusted to 5% CO_2_ and 2.5% O_2_ (Galaxy 170 R incubator, New Brunswick Scientific, Hatfield, UK). To determine clearance from tissues, ACCM-2 solid media was supplemented with 0.5 mM tryptophan, which has been shown to improve recovery of bacteria at low numbers [36].

### 4.5. Inhalation Challenge and Dosing

The challenge was performed using a contained Henderson apparatus controlled by the AeroMP (Aerosol Management Platform) aerosol system (Biaera Technologies L.L.C., Hagerstown, Md, USA) using a 3-jet Collison nebuliser as previously described [15]. Administration of inhalational antibiotic was performed using a Pari eFlow^®^ rapid system (Pari Medical Ltd., West Byfleet, UK) as previously described [35].

### 4.6. Immunological Analysis

Single-cell suspensions of blood (collected pre- and post-challenge), lung, and spleens had contaminating red blood cells lysed by the addition of lysis buffer (BD Biosciences, Wokingham, UK). They were then stained using 3 sets of six or seven of anti-human antibodies conjugated to fluorescent dyes to identify cell phenotypes and activation status by flow cytometry. Lymphocyte stains were antibodies against: CD3 (SP34-2), CD8 (LT8), CD56 (B159), CD69 (FN50), CD20 (Bly1), CD16 (3G8), and TCR-γδ (B1); monocytes/macrophages and neutrophil stains were antibodies against: CD11c (SHCL3), CD14 (M5E2), CD16 (3G8), CD54 (HCD54), CD163 (GHI/61), CD80 (2D10), CD40 (5C3), CD64 (10.1), and HLA-DR (L243) (BD Bioscience, Wokingham, UK; BioLegend, London, UK; Bio-Rad AbD Serotec Limited, Kidlington, UK). Stained cells were fixed for 40 h in a final volume of 4% paraformaldehyde. Samples were analysed on a BD FACSCanto II and cell populations were determined using BD FACSDiva software, v9.0). Whole cells (as determined by the presence of an intact nucleus but not necessarily an intact cell membrane) were detected by nuclear staining, allowing the area of interest to be defined by forward and side scatter. Forward and side scatter were also used to gate areas for detection of lymphocytes (T and B cells), natural killer (NK) cells, macrophages (M0), and neutrophils.

### 4.7. Re-Stimulation Assay

Spleens (aseptically removed at post-mortem) were finely minced using scissors and washed through a 45 μm cell sieve to produce single-cell suspensions with a high proportion of undamaged viable cells. Cells were diluted into L-15 media supplemented with glutamax, 10% foetal calf serum, Penicillin, and Streptomycin (all from Invitrogen, Paisley, UK) to give a cell density of approximately 2 × 10^6^ cells/mL. Previous work has determined that a normal-sized marmoset spleen contains 1 × 10^8^ cells in total. These cells were incubated for 24 h (37 °C without CO_2_) in the presence of one of the following: media only (unstimulated negative control), 2.5 μg/mL ConA (Sigma, Welwyn Garden City, UK. positive control), 1 × 10^7^ cfu/mL Heat Killed Phase I (PhI) *C. burnetii*, or 1:10 dilution of Qvac vaccine (as test antigens). After incubation, the supernatant was stored at −80 °C until it could be measured for the level of IFN-γ expression.

### 4.8. Antibody ELISA

Maxisorb 96-well Elisa plates (Corning, Ewloe, UK) were coated with 1 × 10^8^ cfu/mL of Heat Killed Phase I (PhI) or Phase II (PHII) *C. burnetii* in carbonate coating buffer (Sigma, Welwyn Garden City, UK) overnight at 4 °C. Plasma collected from animals on Day 28 was stored frozen at −80 °C until testing and used at a starting dilution of 1:50. Plasma was diluted 1:2 to obtain an endpoint titre and measured in duplicate. The endpoint was determined as 2 standard deviations above the background.

### 4.9. Cytokine Analysis

Following the downstream processing of tissues extracted from marmosets, plasma and supernatant fluid collected from homogenised lung and spleen tissue were stored at −80 °C. IL-6, IL-1β, MCP-1(CCL2), and RANTES (CCL5) were analysed using antibodies from the BD^TM^ Cytometric Bead Array (CBA, BD Biosciences, Wokingham, UK) human Flex set. IFN-γ, TNF-α, IL-2, and IL-17 were custom-made kits manufactured by BBI Detection Ltd. (Salisbury, UK) using marmoset-specific antibodies produced by U-Cytech Biosciences (Utrecht, The Netherlands). Samples were analysed on a BD FACSCanto II (BD Biosciences, Wokingham, UK) and quantities of bound cytokines were determined using BD FACSDiva software v 9.0 (BD Biosciences, Wokingham, UK).

### 4.10. Histopathological Analysis

Tissues were fixed in 10% neutral buffered formalin and processed for paraffin wax embedding using standard techniques. Thin sections (4 μm) were cut and stained with Haematoxylin and Eosin (H&E) for histopathological analysis. The tissues were examined by light microscopy and evaluated subjectively. The lesion profiles were evaluated and scored from 0 to 4 as follows: 0 = Within normal limits, 1 = Minimal, 2 = Mild, 3 = Moderate, and 4 = Severe.

### 4.11. Liver Enzymes

Blood was collected from the animals pre-challenge and post-mortem, as well as on Days 8 and 14, into Lithium heparin blood tubes. Plasma from heparinised blood was used to assess the liver enzymes, which were analysed using a ‘dry-slide’ technology biochemistry analyser (Catalyst Dx, IDEXX Laboratories Limited, Wetherby, UK).

### 4.12. Statistics

The main hypothesis of this work was to determine whether the antibiotics (Apulmiq and/or doxycycline) would alter disease severity as measured by the temperature response, weight loss, and/or bacterial load. Data was blinded until the analysis was completed. Comparative analysis of bacteriology, histology scores, immunology, and liver enzyme data was performed using one-way ANOVA or Kruskal–Wallis analysis, with data transformed by Log_10_ to ensure normal distribution as appropriate. The suitability of data (or transformed data) for parametric analysis was assessed by examination of the model residuals in QQ and residual plots. Individual one-way analysis for each timepoint was selected due to the dynamic nature of the disease and identification of greater effects at these specific points. The post-tests used were Tukey’s test for one-way ANOVA and Dunn’s for Kruskal–Wallis. The analysis and graphs were prepared using GraphPad PRISM V10 Software. Additional multivariate analysis was performed using the software SPSS V29. Prior to multivariate analysis, some variables required transformation and missing values to be imputed. The marmoset that succumbed to disease was removed from analysis due to the limited data available generated from this animal. All other data was included in all other analyses. The requirement for transformation was assessed using diagnostic plots and several variables were transformed by Log_10_. Where one to three values were missing, these were imputed using a standard regression method that accounted for antibiotic group differences. Ten data points (of a total of 570 data points) needed imputation. The factor analysis used in this study was deployed via a Varimax rotation factor.

## 5. Conclusions

This study confirmed the utility of the model and the resulting readouts and demonstrated the superior protection afforded by doxycycline. In addition, IFN-γ is a useful additional secondary marker. Furthermore, the use of fever and a single blood sample at peak fever to quantify bacteraemia and circulating IFN-γ provides clear indicators of antibiotic success (Figure 10 and Figure 11).

## Figures and Tables

**Table 1:** Key pharmacokinetic data obtained in marmosets following one or more oral doses of doxycycline.

Parameter	Single Dose (10 mg/kg)	Multiple Doses (4 mg/kg)	
	Dose 1	Day 1 (Dose 1)	Day 5 (Dose 5)
**T_max_** (h)	1.1	1.8	1.4
**C_max_** (mg/L)	3.8	0.9	2.4
**t_1/2_** (h)	11.1	5.2	4.0
**AUC** (h.mg/L)	64.9	8.4	17.5
**CL/F** (L.h.kg)	0.2	0.5	0.2
**V_F** (L.kg)	2.5	3.6	1.3
**Absorption rate constant, *k_a_*** (h^−1^)	3.6	1.5	1.9
**Elimination rate constant, *k*** (h^−1^)	0.06	0.13	0.17

## Data Availability

The original contributions presented in this study are included in the article. Further inquiries can be directed to the corresponding author. The contents include material subject to © Crown copyright (2026), Dstl. This information is licensed under the Open Government Licence v3.0. To view this licence, visit https://www.nationalarchives.gov.uk/doc/open-government-licence/. 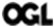 accessed on the 3 August 2026. Where we have identified any third-party copyright information, permission from the copyright holders concerned will need to be obtained. Any enquiries regarding this publication should be sent to: centralenquiries@dstl.gov.uk
